# Living the Sweet Life: How *Liquorilactobacillus hordei* TMW 1.1822 Changes Its Behavior in the Presence of Sucrose in Comparison to Glucose

**DOI:** 10.3390/foods9091150

**Published:** 2020-08-21

**Authors:** Julia Bechtner, Christina Ludwig, Michael Kiening, Frank Jakob, Rudi F. Vogel

**Affiliations:** 1 Lehrstuhl für Technische Mikrobiologie, Technische Universität München (TUM), 85354 Freising, Germany; julia.bechtner@tum.de (J.B.); frank.jakob@tum.de (F.J.); 2 Bavarian Center for Biomolecular Mass Spectrometry (BayBioMS), 85354 Freising, Germany; tina.ludwig@tum.de; 3 Lehrstuhl für Genomorientierte Bioinformatik, Technische Universität München (TUM), 85354 Freising, Germany; michael.kiening@tum.de

**Keywords:** *Liquorilactobacillus hordei*, *Lactobacillus hordei*, dextransucrase, proteomics, exoproteome, water kefir, sucrose, metabolism

## Abstract

*Liquorilactobacillus* (*L.*) *hordei* (formerly *Lactobacillus hordei*) is one of the dominating lactic acid bacteria within the water kefir consortium, being highly adapted to survive in this environment, while producing high molecular weight dextrans from sucrose. In this work, we extensively studied the physiological response of *L. hordei* TMW 1.1822 to sucrose compared to glucose, applying label-free, quantitative proteomics of cell lysates and exoproteomes. This revealed the differential expression of 53 proteins within cellular proteomes, mostly associated with carbohydrate uptake and metabolism. Supported by growth experiments, this suggests that *L. hordei* TMW 1.1822 favors fructose over other sugars. The dextransucrase was expressed irrespectively of the present carbon source, while it was significantly more released in the presence of sucrose (log_2_FC = 3.09), being among the most abundant proteins within exoproteomes of sucrose-treated cells. Still, *L. hordei* TMW 1.1822 expressed other sucrose active enzymes, predictively competing with the dextransucrase reaction. While osmolysis appeared to be unlikely, sucrose led to increased release of a multitude of cytoplasmic proteins, suggesting that biofilm formation in *L. hordei* is not only composed of a polysaccharide matrix but is also of proteinaceous nature. Therefore, our study highlights the intrinsic adaptation of water kefir-borne *L. hordei* to sucrose-rich habitats and provides fundamental knowledge for its use as a starter culture in plant-based food fermentations with in situ dextran formation.

## 1. Introduction

Especially in the food sector, the new health conscious nature of the western population nowadays demands innovative approaches in the production of foods with sophisticated nutritional and techno-functional properties [1,2]. While the demand for animal products is decreasing, plant-based alternatives are attracting simultaneously more attention [3]. Thus, especially in the manufacturing of fermented foods, this requires the application of starter cultures that are adapted to plant material fermentation [4]. However, fermented fruit and vegetable products already have a long history and are only waiting to be explored for the occurrence of microorganisms that may be exploited in innovative food manufacturing processes.

As such, water kefir is a traditional fermented beverage that is made from dried fruits, lemon slices and sucrose, and its microbiota is therefore perfectly adapted to metabolize plant-derived nutrients [5,6]. The beverage itself is believed to exhibit antibacterial, anti-inflammatory, anti-allergic, anti-hyperglycemic and further beneficial health effects, while the microbiota is considered as historically safe for consumption [7,8,9,10,11]. The kefir granules, which harbor a complex consortium of mainly yeasts, lactic acid bacteria (LAB), acetic acid bacteria and bifidobacteria, thus host an interesting reservoir for microorganisms, which can be applied in innovative plant-based food and beverage fermentations [12]. While some water kefir-borne LAB were already shown to exhibit probiotic potential [13,14], the production of exopolysaccharides with different techno-functional properties harbors additional application potential [15,16]. During water kefir fermentation, the main exopolysaccharide produced by LABs is dextran [17]. Dextrans are mainly α-1,6-linked glucans that are specifically formed from sucrose by extracellular enzymes, namely dextransucrases [18,19]. These polysaccharides exhibit different techno-functional properties depending on the basic dextran type, concentration and macromolecular structure that are inter alia influenced by the type of dextransucrase, substrate concentration, pH, temperature, salt concentration and other factors [16,20,21,22,23]. During water kefir fermentation, the inhabiting LABs naturally produce different types of dextrans. While gel-forming dextrans build up the water-insoluble network of the kefir granules, others are responsible for the turbid characteristics of the supernatant [16,17,24,25,26]. Dextrans not only show texturizing, emulsifying and cloud-forming characteristics but are additionally studied for their potential as prebiotics [27,28,29,30,31]. Moreover, the addition of sucrose as a carbon source for food fermentation processes would enable in situ dextran synthesis and thereby the manufacture of “clean label” products with improved properties [16,32,33].

*Liquorilactobacillus* (*L.*) *hordei* (formerly *Lactobacillus hordei* [34]) was first isolated from malted barley, indicating the species’ adaptation to live in plant habitats [35]. Until now, it was, however, more frequently isolated from water kefir, where it was found to occur in high abundance [12]. To survive in this environment, efficient sucrose metabolism is essential, as sucrose is the main source of energy in water kefir, which is otherwise poor in nutrients. A comparative genomic analysis of water kefir isolate *L. hordei* TMW 1.1822 and the type strain counterpart, isolated from malted barley, highlighted the adaptation of the water kefir isolate to sucrose-rich habitats, exhibiting additional enzymes and transporters for the efficient uptake and metabolism of sucrose and fructose [36]. This genomic difference also applied for the dextransucrase, encoded by the water kefir isolates *L. hordei* TMW 1.1822 and TMW 1.1907, respectively, which ensures efficient extracellular sucrose degradation upon simultaneous formation of a water-soluble dextran [24,36]. This characteristic of the water kefir isolates recently gained interest in the fermentation of fruit juice substrates [16]. However, it was shown that *L. hordei* accumulates its dextransucrase intracellularly and solely releases it in the presence of sucrose [21,24]. This indicates that sucrose is not only a growth substrate but may also induce changes in the exoproteome of this microorganism. As *L. hordei* is a starter culture candidate for more plant-based food fermentations besides water kefir, exploration of its physiological response to this carbohydrate is essential. In this study, we therefore studied the behavior of *L. hordei* TMW 1.1822 in the presence of sucrose, applying a label-free proteomic approach, physiological tests and measurements of consumed and produced metabolites, comparing growth in glucose versus sucrose.

## 2. Materials and Methods

### 2.1. Strains, Media and Growth Conditions

Cryo-conserved *L. hordei* strains isolated from water kefir (TMW 1.1817, TMW 1.1821, TMW 1.1822, TMW 1.1907, TMW 1.2375, TMW 1.2376, TMW 1.2377) and the type strain isolated from malted barley (DSMZ 19,519 = TMW 1.2353) were recovered by static incubation in 15 mL liquid modified MRS medium [37] (10 g/L soy peptone, 10 g/L meat extract, 5 g/L yeast extract, 25 g/L glucose, 1 g/L Tween80, 2 g/L dipotassium phosphate, 5 g/L sodium acetate, 2 g/L di-ammonium citrate, 0.2 g/L magnesium sulfate, 0.05 g/L manganese sulfate, pH adjusted to 6.2) in closed vessels at 30 °C. Depending on the performed experiment, incubation times varied and glucose was replaced by other carbohydrates. To determine viable cell counts (cfu/mL), 100 µL of appropriate dilutions in Ringer’s solution (Merck, Darmstadt, Germany) were spread on MRS agar plates (1.5%) with sterile glass beads (2.7 mm, Carl Roth, Karlsruhe, Germany) and incubated at 30 °C for 48 h.

### 2.2. Proteomic Analysis

#### 2.2.1. Experimental Setup

To investigate the proteomic response to sucrose intracellularly and extracellularly, 5 × 15 mL precultures (five biological replicates) of *L. hordei* TMW 1.1822 were prepared in MRS medium as described above (Section 2.1) and used to inoculate 5 × 50 mL cultures in MRS (25 g/L glucose), with a final OD_600nm_ of 0.1. The cultures were grown to mid-exponential growth phase (~OD_600nm_ 2.0, determined in preliminary experiments), which had given good results in previous experiments [33,38]. In total, 15 mL of each biological replicate was distributed to two 15 mL reaction vessels, pelletized (3000× *g*, 4 °C, 5 min) and washed once with fresh MRS medium. After a second centrifugation step, cell pellets were resuspended in 15 mL of medium supplemented with either glucose or sucrose (25 g/L each). After incubation for 2 h at 30 °C, a 100 µL sample was taken for determination of viable cell counts, and cultures were subsequently centrifuged (3000× *g*, 4 °C, 5 min). Supernatants were collected and frozen at −20 °C for subsequent experiments and exoproteome analysis, while cells were washed twice with 10 mL Ringer’s solution (4 °C) and following centrifugation. Finally, cell pellets were immediately frozen with liquid nitrogen and stored at −80 °C for subsequent proteomic analysis.

#### 2.2.2. Peptide Preparation, Separation and Mass Spectrometry

Cellular lysate samples: Sample preparation and measurements of cellular lysate samples were done as described by Prechtl et al. [33]. Therefore, cell pellets were resuspended in 900 µL lysis buffer (8 M urea, 5 mM ethylenediaminetetraacetic acid (EDTA) disodium salt, 100 mM NH_4_HCO_3_, 1 mM dithiothreitol (DTT) in water, pH 8.0) supplemented with 10× solution SIGMA*FAST*™ protease inhibitor cocktail (Sigma-Aldrich, St. Louis, MO, USA) according to the manufacturer´s instructions. Subsequently, cells were disrupted mechanically using 400 mg glass beads (G8772, 424–600 µm, Sigma, Germany) for 10 min at 4 °C. Total protein concentrations of the lysates were determined using the Coomassie (Bradford, UK) Protein Assay Kit (ThermoFisher Scientific, Rockford, IL, USA), according to the manufacturer´s instructions. In total, 100 µg of protein extract of each sample was used for in-solution digestion. Proteins were reduced (10 mM DTT, 30 °C, 300 rpm, 30 min) and carbamidomethylated (55 mM chloroacetamine, 30 min, RT, in the dark) and subsequently diluted 6× with freshly prepared NH_4_HCO_3_ solution (0.05 M). Afterwards, 1 µg of trypsin (trypsin to protein ration 1:100) was added and samples were incubated for 4 h at 30 °C and 300 rpm. After a second addition of trypsin (same amount), samples were incubated overnight at 30 °C and 300 rpm. Protein digestion was stopped using 1% (*v*/*v*) formic acid. Digested protein samples were desalted using C18 solid phase extraction with Sep-Pak columns (Waters, Milford, MA, USA, WAT054960), according to the manufacturer´s instructions. Finally, purified peptide samples were dried in a SpeedVac and re-dissolved in an aqueous solution of acetonitrile (2%) and formic acid (0.1%) at a final concentration of 1 µg/µL.

Exoproteome samples: Exoproteomes were prepared as described by Heinze et al. [39]. Briefly, 30 µL of each supernatant sample and un-inoculated MRS medium (negative control) were mixed with lithium dodecyl sulfate (LDS) sample buffer (ThermoFisher Scientific, Waltham, MA, USA), reduced with 25 mM DTT, heated for 10 min at 95 °C and alkylated with 55 mM chloroacetamine. Samples were applied on a 4–12% NuPAGE gel (ThermoFisher Scientific, Waltham, MA, USA) and run for around 1 cm to concentrate all proteins. In-gel digestion was performed according to standard procedures described by Shevchenko et al. [40]. The obtained peptides were dried and re-dissolved as stated above.

Nano-flow LC-MS/MS measurements were performed on a Fusion Lumos Tribrid mass spectrometer (Thermo Fisher Scientific) equipped with an Ultimate 3000 RSLCnano system. For each analysis, 0.1 µg of cellular peptides or 0.5 µg of exoproteome peptides was delivered to a trap column (ReproSil-pur C18-AQ, 5 μm, Dr. Maisch, 20 mm × 75 μm, self-packed) at a flow rate of 5 μL/min in 100% solvent A (0.1% formic acid in HPLC grade water). After 10 min of loading, peptides were transferred to an analytical column (ReproSil Gold C18-AQ, 3 μm, Dr. Maisch GmbH, Ammerbuch-Entringen, Germany, 450 mm × 75 μm, self-packed) and separated using a 50 min gradient from 4% to 32% of solvent B (0.1% formic acid in acetonitrile and 5% (*v*/*v*) DMSO) at a 300 nL/min flow rate. Both nanoLC solvents contained 5% (*v*/*v*).

The Fusion Lumos Tribrid mass spectrometer was operated in data-dependent acquisition and positive ionization mode. MS1 spectra (360–1300 m/z) were recorded at a resolution of 60,000 using an automatic gain control (AGC) target value of 4 × 10^5^ and maximum injection time (maxIT) of 50 ms. After peptide fragmentation using higher energy collision induced dissociation (HCD), MS2 spectra (200–2000 m/z) of up to 20 precursor peptides were acquired at a resolution of 15.000, with an automatic gain control (AGC) target value of 5 × 10^4^ and maximum injection time (maxIT) of 22 ms. The precursor isolation window width was set to 1.3 m/z and normalized collision energy to 30%. Dynamic exclusion was enabled with 20 s exclusion time (mass tolerance +/−10 ppm). Peptide precursors that were singly charged, unassigned or with charge states >6+ were excluded for fragmentation.

#### 2.2.3. Data Availability

All LC-MS/MS data files and MaxQuant output files have been deposited in the ProteomeXchange Consortium (http://proteomecentral.proteomexchange.org) via the PRIDE partner repository with the dataset identifier PXD020664.

#### 2.2.4. Protein Identification and Quantification

Peptides and proteins were identified and quantified using the MaxQuant software (v. 1.6.3.4) [41] with its built-in search engine, Andromeda [42]. MS2 spectra were searched against all protein sequences predicted for the genome of *L. hordei* TMW 1.1822 (GenBank CP018176–CP018179) supplemented with common contaminants (built-in option in MaxQuant). Trypsin/P was specified as proteolytic enzyme. Precursor tolerance was set to 4.5 ppm and fragment ion tolerance to 20 ppm. Results were adjusted to 1% false discovery rate (FDR) on peptide spectrum match (PSM) level and protein level, employing a target-decoy approach using reversed protein sequences. The minimal peptide length was defined as 7 amino acids; the “match-between-run” function was disabled. Carbamidomethylated cysteine was set as fixed modification and oxidation of methionine and N-terminal protein acetylation as variable modifications. To compare relative protein abundances between samples, label-free quantification (LFQ) was performed. Additionally, also intensity-based absolute quantification (iBAQ) was enabled, which provides an estimate of the absolute protein abundance and thereby a proportional quantification unit for the abundance of different proteins within one sample.

MaxQuant output files were further processed and statistically analyzed using Perseus software (v. 1.6.14.0) [43]. Only proteins that were identified in four out of five biological replicates in at least one group (glucose or sucrose) were considered. Missing values were imputed from a normal distribution (width: 0.2; down shift: 1.8). The obtained log_2_-transformed LFQ intensities were used for a Student’s T-test analysis with a permutation-based FDR of 0.01 and S0 of 0.1. Absolute protein abundances at a certain condition were estimated using the averaged log_10_-transformed iBAQ intensities that were ranked in descending order for each group.

Additionally, MS intensities of lysates and exoproteomes were compared in order to prioritize extracellular proteins that ended up in the medium due to some form of active biological process rather than cell death or cell lysis. Therefore, z-scores of log_10_-transformed iBAQ intensities were calculated in Perseus software using the average and standard deviation over all iBAQ intensities within each group (matrix access: column). The obtained z-scores were statistically analyzed by Student’s T-test analysis, with a permutation-based FDR of 0.01 and S0 of 0.1. Thereby, normalized iBAQ intensities of exoproteomes and lysates of each condition (glucose or sucrose) were compared with each other. Proteins with a difference between z-score (exoproteome) minus z-score (cellular proteome) higher or equal than 2.0 were considered as being released with high confidence.

#### 2.2.5. GO-Enrichment Analysis

Gene ontology (GO) enrichment analysis was performed on the basis of the statistically analyzed proteomic datasets obtained by the analysis of LFQ intensities (see Section 2.2.4), using the topGO package (v. 2.40.0) running in R Studio (v. 4.0.2) [44]. Genes were filtered according to their significance as obtained during statistical analysis in Perseus and their up- or down-regulation in glucose and sucrose-treated cells (see Section 2.2.4). Statistical significance of enriched GO terms was indicated by a Fisher’s exact *p*-value ≤ 0.05 (classicFisher).

### 2.3. Analysis of Culture Supernatants of Glucose and Sucrose-Treated Cells

#### 2.3.1. Quantification of Sugar Consumption and Acid Formation

Consumption of glucose and sucrose as well as formation of organic acids was measured in the exoproteome samples obtained for further proteomic analysis (see Section 2.2.1). Therefore, concentrations were determined using a HPLC system (Dionex Ultimate 3000, ThermoFisher Scientific, Waltham, MA, USA) coupled to a refractive index (RI) detector (Refractomax ERC, Germany). For organic acid quantification, 1 mL of sample was mixed with 50 µL perchloric acid (70% (*v*/*v*)), mixed thoroughly and incubated overnight at 4 °C. Afterwards, samples were centrifuged at 13,000× *g* for 30 min at 4 °C. Prior to application on the HPLC system, all samples were filtered (0.2 µm nylon filters, Phenomenex, Aschaffenburg, Germany) to remove any aggregates. Subsequently, 20 µL of each filtered sample was injected into the HPLC system. Sugars were measured using a Rezex RPM Pb^2+^ column (Phenomenex, Aschaffenburg, Germany) at a flow rate of 0.6 mL/min (85 °C), using filtered (0.2 µm) deionized water as eluent. Organic acids were measured with a Rezex ROA H^+^ column (Phenomenex, Aschaffenburg, Germany) at a flow rate of 0.7 mL/min (85 °C), with 2.5 mM H_2_SO_4_ (prepared with filtered deionized water) as eluent. Identification and quantification of sugars and organic acids was performed according to external standards using the Chromeleon software (v. 6.8; ThermoFisher Scientific (Dionex), Waltham, MA, USA).

#### 2.3.2. SDS-PAGE, Staining and Zymogram

Exoproteome samples that were used for further proteomic analysis (see Section 2.2.1) were analyzed by vertical SDS-PAGE, carried out in a Mini-PROTEAN Tetra Cell Electrophoresis System (Bio-Rad Laboratories, Hercules, CA, USA). A separation gel (10% (*w*/*v*)) with a stacking gel (4% (*w*/*v*)) were used. Prior to loading on the gel, protein samples were diluted in 2× Laemmli buffer (Sigma-Aldrich, St. Louis, MO, USA) and denatured at 90 °C for 10 min. Separation was initially started at 100 V for 10 min and continued at 150 V for 60 min using a Power Pack 3000 unit (Bio-Rad laboratories, Hercules, CA, USA). Visualization of proteins was performed by silver staining, as described by Blum et al. [45]. To detect possible cell wall degrading enzymes, a zymogram analysis was performed, as described by Lepeuple et al. [46], with some changes. In brief, the separation gel was supplemented with 25% (*v*/*v*) of bacterial substrate (cells of *L. hordei* TMW 1.1822 or *Micrococcus* (*M.*) *luteus* TMW 2.96) prior to protein loading. Instead of 2× Laemmli buffer, a 2× native PAGE sample buffer (60 g/L Tris Base, 40 g/L sodium dodecyl sulfate (SDS), 20% (*v*/*v*) glycerol (87%), traces of bromophenol blue) was used to prepare protein samples. The bacterial substrates were prepared as follows. *L. hordei* TMW 1.1822 was precultured as stated above (see Section 2.1) and subsequently used to inoculate 50 mL of liquid MRS medium to an OD_600nm_ of 0.1. Cells were grown to mid-exponential growth phase, as stated above (see Section 2.2.1), and harvested by centrifugation (3000× *g*, 4 °C, 5 min). Afterwards, the obtained cell pellet was washed once with 5 mL of Tris buffer (20 mM Tris, 100 mM NaCl, pH 7.4, 4 °C). After centrifugation, cells were resuspended in 4 mL Tris buffer (1.5 M Tris, pH 8.8). Prior to incorporation into the gel, the bacterial substrate was incubated at 95 °C for 10 min. Harvesting of *M. luteus* TMW 2.96 cells was done as for *L. hordei* TMW 1.1822, but cultures were directly inoculated from cryo cultures and subsequently grown overnight at 37 °C and 200 rpm in LB medium (5 g/L yeast extract, 10 g/L tryptone, 5 g/L NaCl).

Following SDS-PAGE, the gel was washed twice in deionized water at room temperature on a shaker for 30 min. Afterwards, it was transferred to renaturing buffer (20 mM Tris, 50 mM NaCl, 20 mM MgCl_2_, 0.5% Triton X-100, pH 7.4) and incubated for 30 min. Subsequently, the gel was transferred into fresh renaturing buffer and incubated overnight at 30 °C. To improve the visibility of lytic zones, the gel was incubated in staining solution (1 g/L methylene blue, 0.1 g/L KOH) for 2 h at room temperature. Lytic zones appeared as clear bands against blue background.

### 2.4. Determination of Growth Parameters in Different Sugars

Cells of *L. hordei* TMW 1.1822 were pre-cultivated as described in Section 2.1. In 96-well plates (Sarstedt, Nümbrecht, Germany), 250 µL of liquid MRS medium containing either glucose, sucrose, fructose or a mixture of glucose and fructose (12.5 g/L each) was inoculated to OD_600nm_ 0.1 and overlaid with 50 µL paraffin oil to prevent the cultures from desiccating. Cell growth was monitored by OD_600_ measurement every 30 min for 30 h at 30 °C in a SPECTROstar Nano Platereader (BMG Labtech, Ortenberg, Germany). Plates were shaken at 400 rpm for 30 s prior to each measurement. Maximum growth rates (µ_max_) and time spans of lag phases were determined using the grofit package for RStudio (v. 3.3.3), as described in Kahm et al. [47]. The same MRS media were used to continuously monitor acidification during cell growth in the iCinac system (AMS, Frépillon, France) at 30 °C for 30 h.

### 2.5. Screening for Dextransucrases in Other L. hordei Strains by PCR

All *L. hordei* strains were incubated as stated above and harvested after 24 h. Therefore, 4 mL of the liquid cultures was pelleted by centrifugation (4000× *g*, 5 min, 4 °C) and washed once with TE buffer (10 mM Tris-HCl, 10 mM EDTA, pH 8.0). Subsequently, DNA was isolated using the E.Z.N.A™ Bacterial DNA Kit (Omega Bio-tek, Doraville, GA, USA), according to the manufacturer´s instructions, but with a prolonged incubation time of 2 h for cell lysis. DNA samples were stored at −20 °C until further investigation.

To screen for dextransucrases in *L. hordei* strains, the prepared DNA samples were applied for PCR analysis using the *Taq* DNA CORE Kit 10 (MP Biomedicals, Irvine, CA, USA). Therefore, 2.5 µL *Taq* 10× buffer with MgCl_2_, 0.5 µL dNTPs (10 mM each), 0.25 µL *Taq*-polymerase (5 U/µL), 18.75 µL dest. H_2_O, 1 µL forward and 1 µL reverse primer (50 mM each) and 1 µL of a DNA sample were applied. Two different sets of primers were used (see Table S1). The reaction was carried out on a Mastercycler^®^ Gradient (Eppendorf, Hamburg, Germany). Denaturation was performed for 1 min at 95 °C, followed by an annealing step for 45 s (for temperature see Table 1) and an elongation step for 1 min at 72 °C. After 28 cycles, the final elongation was done at 72 °C for 10 min. The obtained PCR products were mixed with 6× loading dye (ThermoFisher, Rockford, IL, USA) and subsequently applied for agarose (1% (*w*/*v*)) gel electrophoresis in TAE buffer (40 mM Tris, 20 mM acetic acid, 1 mM EDTA, pH 8.2) for 90 min at 100 V in a PeQlab electrophoresis chamber (PeQlab Biotechnologie GmbH, Erlangen, Germany). PCR bands were subsequently stained with dimidiumbromide solution (Carl Roth, Karlsruhe, Germany).

## 3. Results

### 3.1. Proteomic Analysis of Cell Lysates and Exoproteomes

#### 3.1.1. Differential Proteomics of Cell Lysates

**Figure 1:**
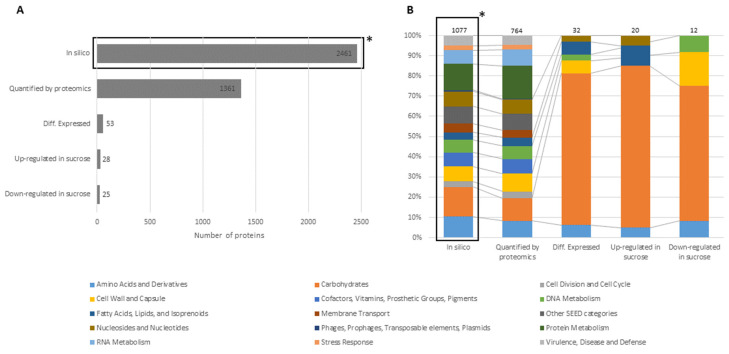
Comparison of the putative functional proteome (in silico) with protein sub-groups obtained by MS intensity statistical analysis of cellular proteomes. (**A**) Total protein counts of in silico predicted proteins, proteins quantified by proteomics (detected in four out of five replicates of at least one group), differentially expressed proteins and up- and down-regulated proteins in sucrose. (**B**) Corresponding SEED category distributions. The SEED subsystem proteome coverage was around 44%. (*) Derived from Di Xu et al. [36].

In our previous studies, *L. hordei* TMW 1.1822 was shown to exhibit 2461 proteins predicted from its genome [36]. In the current study, 1361 proteins were quantified during proteomic analysis (see Figure 1A), which matched the filtering criteria.

The differential expression analysis revealed that 53 of these proteins were significantly differentially expressed in the presence of sucrose compared to glucose (Figure 1A, Table 1). As shown in Figure 1B, solely 30 of the 53 differentially expressed proteins were categorized into subsystems by the SEED-based annotation service RAST [48], of which ~80% belonged to the carbohydrate metabolism category. This included several gene clusters related to the uptake and metabolism of sucrose, fructose, glucose, mannitol, glycerol and beta-glucosides.

**Figure 2:**
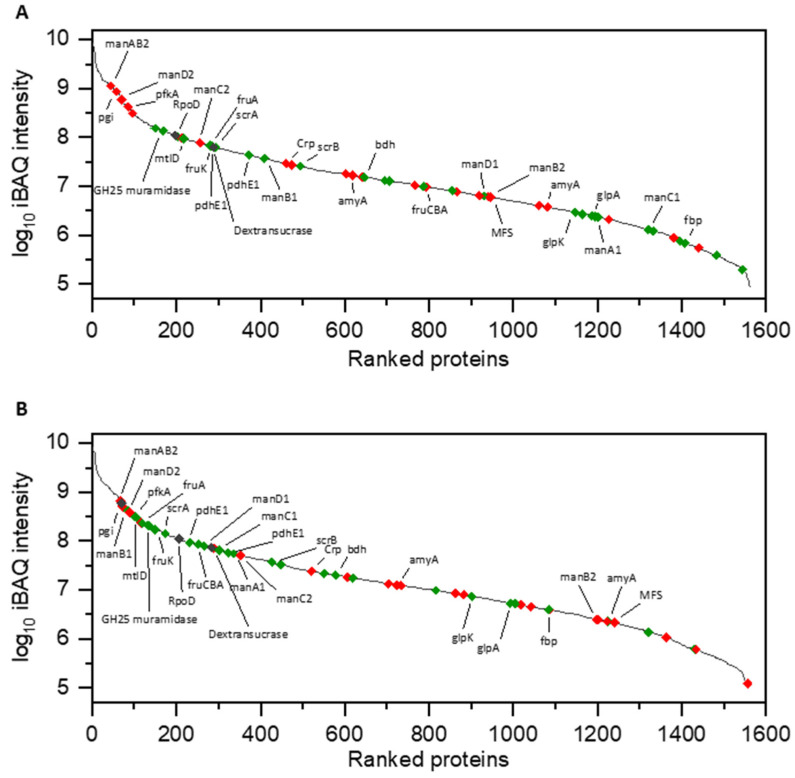
MS intensity ranking of identified proteins in cellular proteomes of cultures treated with glucose (**A**) and sucrose (**B**). Red color = significantly down-regulated in sucrose, green color = significantly up-regulated in sucrose (see Table 1). *amyA* = glucohydrolase; *manA-D* = PTS*^man^*; *pgi* = glucose-6-phosphate isomerase; *pfkA* = 6-phosphofructokinase; *mtlD* = mannitol-1-phosphate 5-dehydrogenase; *fruK* = 1-phosphofructokinase; *fruA* + *fruCBA* = PTS*^fru^*; *scrA* = PTS*^scr^*; *pdhE1* = pyruvate dehydrogenase E1 subunits; *Crp* = catabolite repression protein; *scrB* = sucrose-6-phosphate hydrolase; *bdh* = butanediol dehydrogenase; *MFS* = sucrose-specific MFS-transporter; *glpA* = glycerol-3-phosphate dehydrogenase; *glpK* = glycerolkinase; *fbp* = fructose-1,6-bisphosphatase.

Regarding sucrose metabolism, a sucrose-specific phosphotransferase system (PTS*^scr^*) (*scrA*, Table 1 #14) was up-regulated (log_2_ fold change (FC) = 1.04) together with sucrose-6-phoshate hydrolase (*scrB*, E.C. 3.2.1.26, Table 1 #13) (log_2_ FC = 0.52) in sucrose-treated cells. By contrast, a sucrose-specific transporter of the major facilitator superfamily (MFS) was significantly down-regulated in the presence of sucrose, showing the highest negative log_2_ FC (=−1.12) among all differentially expressed proteins. According to MS intensity ranking (Figure 2), the PTS*^scr^* system was of high abundance in sucrose and glucose, respectively, while the MFS transporter appeared to be of low abundance in both conditions.

Apart from this, the expression of the gene encoding the *L. hordei* dextransucrase (BSQ49_11535, E.C. 2.4.1.5) was not significantly influenced by the present carbon source. Additionally, the analysis of the respective MS intensities revealed that this enzyme was present in relatively high amounts within the cellular proteomes of both conditions (Figure 2).

Regarding fructose and mannitol metabolism, a PTS*^fru^* system (*fruCBA*, Table 1 #50 + 52) for the uptake and simultaneous 1-phosphorylation of fructose and mannitol was up-regulated (log_2_ FC = 1.64–2.73) in sucrose. Additionally, the mannitol-1-phosphate 5-dehydrogenase (*mtlD*, E.C. 1.1.1.17, Table 1 #49) (log_2_ FC = 1.60), belonging to the same gene cluster, and 1-phosphofructokinase (*fruK*, E.C. 2.7.1.56, Table 1 #5) (log_2_ FC = 1.22) were significantly up-regulated in the presence of sucrose. Moreover, two PTS*^man^* systems (Table 1 *manA-D*1 #37–40 and *manA-D*2 #44–47) for the uptake and simultaneous 6-phosphorylation of fructose and other monosaccharides like mannose were significantly differentially expressed. One of these PTS systems (*manA-D*1) showed the highest positive log_2_ FC (= 3.24–3.85) among all differentially expressed proteins in sucrose. While exhibiting MS intensities in the mid-range of the proteome in the presence of glucose, this PTS*^man1^* system became one of the most abundant proteins, when cells were incubated in sucrose (Figure 2). Although being one of the most abundant proteins in glucose and sucrose, respectively, the other PTS*^man2^* system (*manA*-*D*2) was significantly down-regulated in sucrose (log_2_ FC = −0.70 to −0.79). Simultaneously, 6-phosphofructokinase (*pfkA*, E.C. 2.7.1.11, Table 1 #22) was significantly down-regulated (log_2_ FC = −0.46). Interestingly, the enzyme fructose-1,6-bisphosphatase (*fbp*, E.C. 3.1.3.11, Table 1 #34), catalyzing the adverse reaction, was significantly up-regulated (log_2_ FC = 1.90) in the presence of sucrose.

Regarding the initial glucose metabolism, glucose-6-phosphate isomerase (*pgi*, E.C. 5.3.1.9, Table 1 #26) was significantly down-regulated in sucrose (log_2_ FC = −0.75), while showing high MS intensities in both conditions (Figure 2).

Regarding the final steps of carbohydrate metabolism, the E1 subunit of the pyruvate dehydrogenase complex (*pdhE1* E.C. 1.2.4.1, Table 1 #2 + 3) (log_2_ FC = 0.30–0.36) and butanediol dehydrogenase (*bdh*, E.C. 1.1.1.4, Table 1 #41) (log_2_ FC = 0.35) appeared to be significantly up-regulated in sucrose.

Four proteins involved in the transport and metabolism of glycerol, namely an ABC transporter specific for glycerol-3-phosphate (Table 1 #42 + 43) (log_2_ FC = 1.12–1.42), glycerolkinase (*glpK*, E.C. 2.7.1.30, Table 1 #9) (log_2_ FC = 0.95) and glycerol-3-phosphate dehydrogenase (*glpA*, E.C. 1.1.5.3, Table 1 #10) (log_2_ FC = 0.75) were significantly up-regulated in sucrose.

Apart from carbohydrate metabolism, a putative GH25 muramidase (Table 1 #53), likely involved in cell envelope remodeling, cell division or lysis, was found to be significantly up-regulated in sucrose (log_2_ FC = 0.51).

All MS intensities are listed in a supplementary Excel file (Table S2).

#### 3.1.2. Differential Proteomics of Exoproteomes

**Figure 3:**
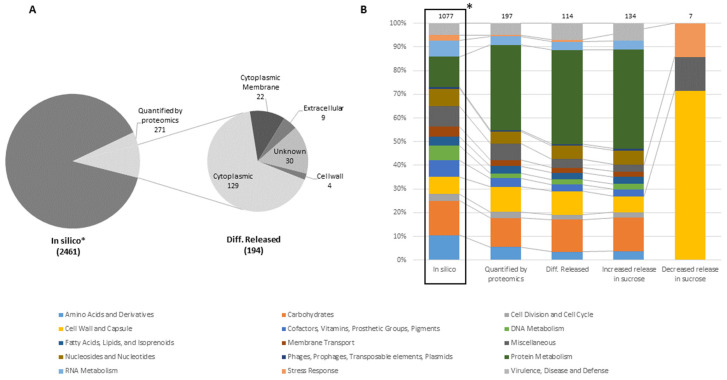
Analysis of the exoproteomes of glucose and sucrose-treated cells. (**A**) Comparison of the putative functional proteome (in silico) using the predicted subcellular localization of proteins, detected in the exoproteomes using proteomics; (**B**) Comparison of the SEED category distributions of the putative functional proteome (in silico), the quantified exoproteomes detected by proteomics (detected in four out of five replicates of at least one group), the differentially released proteins and proteins that were significantly more or less released in the presence of sucrose. The SEED subsystem proteome coverage was around 44%. (*) Derived from Di Xu et al. [36].

Solely few proteins (maximum of eight) were found in un-inoculated medium samples (negative control) that could be assigned to the putative functional proteome of *L. hordei* TMW 1.1822, which may result from carry-over during preparative SDS-PAGE. While 1361 proteins could be quantified in the proteomes of cell lysates, solely 271 proteins were specifically detected in culture supernatants assigned as exoproteomes (Figure 3A).

In total, 194 of these proteins were significantly differentially released in the presence of sucrose compared to glucose. Moreover, 162 of these proteins were subjected to increased release in sucrose, around five times more than proteins affected by decreased release (= 32) in sucrose. The majority of proteins that were increasingly released belonged to the protein metabolism SEED category (~42%), followed by the carbohydrate metabolism category (~14%) (Figure 3B). Around 80% of the proteins increasingly released in the presence of sucrose were assigned to the category of intracellular proteins (Figure 3A). As such, 27 ribosomal proteins, two lactate dehydrogenases (*ldh*, E.C. 1.1.1.27), two proteins of a F0F1-ATPase gene cluster (*atpA, atpD*, E.C. 3.6.3.14), four elongation factors (G, Ts, Tu, IF-3) and the housekeeping proteins *DnaK* and *GroL* appeared to be significantly more released in the presence of sucrose (Table S2). This led to significant GO enrichment of proteins involved in translation (GO: 0006412, *p* = 0.0375) among proteins that were increasingly released in the presence of sucrose (Table 2).

Moreover, a putative beta-fructosidase (*sacC*, BSQ49_09800) was found to be significantly (log_2_ FC = 4.75) more released in the presence of sucrose. The protein was previously thought to be incomplete in the genome and thus not functional in *L. hordei* TMW 1.1822 [24]. Using the ExPASy online translation tool (https://web.expasy.org/translate), translation of the corresponding genomic region and subsequent analysis of conserved domains by the NCBI conserved domain viewer (https://www.ncbi.nlm.nih.gov/Structure/cdd/wrpsb.cgi) revealed the presence of a cell wall anchor and domains equal to beta-fructosidases that may also cleave fructans. However, MS intensity analysis showed that this enzyme was of low abundance compared to the other extracellular sucrose active enzyme, dextransucrase (BSQ49_11535, E.C. 2.4.1.5), which was also significantly more released in the presence of sucrose (log_2_ FC = 3.0).

The majority (~71%) of proteins that were decreasingly released in the presence of sucrose belonged to the cell wall and capsule SEED category, including two flagellum associated murein hydrolases (*flgJ,* BSQ49_00575 and BSQ49_10650) and two putative peptidoglycan endopeptidases containing an NlpC/P60 domain (BSQ49_02730 and BSQ49_11125) (Figure 3B). However, solely seven out of 32 significantly less released proteins were annotated by the SEED-based annotation server, RAST. To complete this analysis, GO analysis revealed the enrichment of flagellar proteins (GO: 0001539, *p* = 8.9 × 10^−7^) among decreasingly released proteins in the presence of sucrose (Table 2).

All MS intensities are listed in a supplementary Excel file (Table S2).

#### 3.1.3. Comparison of Proteomic States of Cell Lysates and Exoproteomes

**Figure 4:**
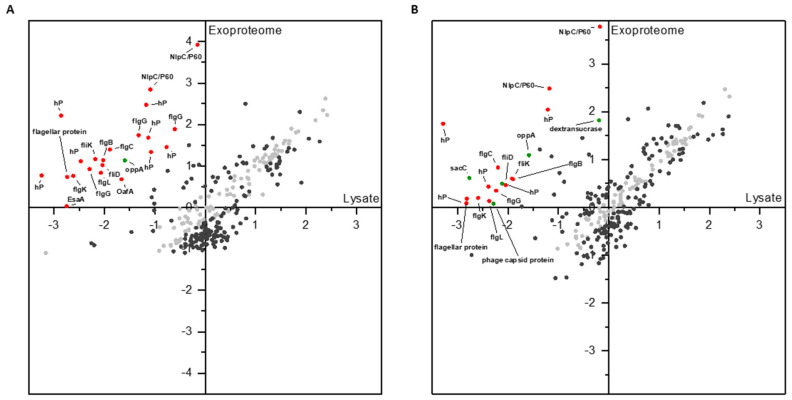
Comparison of abundances of proteins identified in cellular and extracellular proteomes in cultures incubated in glucose (**A**) and sucrose (**B**). All log_10-_transformed iBAQ intensities were normalized by z-scoring. Light gray = proteins with similar relative abundance in cellular and extracellular proteomes; dark gray, red or green color = proteins of significantly different relative abundancy (FDR ≤ 0.01, S0 = 0.1); red and green color = proteins subjected for directed release with high confidence (z-score difference exoproteome − cellular proteome ≥ 2.0); red color = proteins decreasingly released in the presence of sucrose; green color = proteins increasingly released in the presence of sucrose (Table S2). hP = hypothetical protein; *flgB, flgC*, *flgG, flgK, fliD, fliK* = flagellar proteins; *oppA* = peptide ABC transporter substrate-binding protein; *OafA* = acetyltransferase; *EsaA* = type VII secretion protein; *sacC* = putative beta-fructosidase.

As the majority of proteins identified in the exoproteome of both conditions (glucose and sucrose) belonged to cytoplasmic proteins, we examined the quantitative correlation of the exoproteomes and the cellular proteomes in order to identify proteins that are significantly more concentrated in the exoproteome and therefore most likely proteins that were actively secreted. Indeed, it could be shown that at least some of the proteins found in the extracellular milieu in glucose or sucrose-treated cells exhibited comparable MS intensities relative to other proteins within the respective exoproteome or lysate (Figure 4). However, the abundance of most of the proteins within the exoproteomes (glucose ~63%; sucrose ~66%) was significantly different from the abundance within lysates at the applied statistical parameters (FDR ≤ 0.01; S0 = 0.1).

Due to the high amounts of these proteins, solely proteins that were subjected for directed release with high confidence (z-score difference exoproteome minus lysate ≥ 2.0) will be discussed further. In sucrose-treated cells, 19 proteins could be assigned to this “high confidence” category, while in glucose-treated cells, 22 proteins were found. Although being less released in the presence of sucrose, the majority of these proteins were flagellum-related in both conditions, respectively (Figure 4). This also applied for the proteins exhibiting an NlpC/P60 domain. However, solely in sucrose-treated cells, the dextransucrase, as well as the putative beta-fructosidase (*sacC*), were identified among these high confidence proteins subjected to directed release (Figure 4B).

### 3.2. SDS-PAGE and Zymogram Analysis of the Exoproteomes of Cells Treated with Glucose or Sucrose

**Figure 5:**
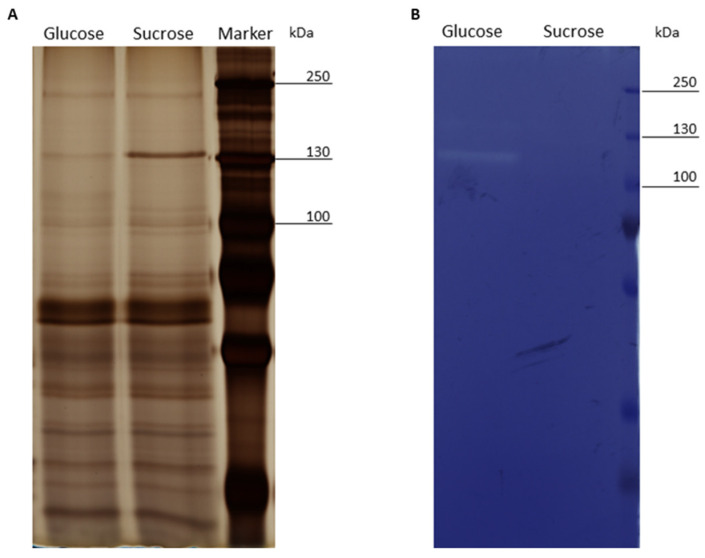
SDS-PAGE analysis of culture supernatants of cells incubated in either glucose or sucrose with subsequent silver staining (**A**) and zymogram analysis for the detection of lytic enzymes on gels containing dead cells of *M. luteus* TMW 2.96 (**B**).

As the proteomic experiment revealed that the majority of proteins found in the supernatants of the cultures were annotated as cytoplasmic proteins, especially after incubation in sucrose-supplemented medium, the exoproteomic samples were subjected to SDS-PAGE and subsequent silver staining. The protein bands appeared well separated, while protein band patterns were highly similar for exoproteomes of glucose and sucrose-treated cells (Figure 5A).

Proteomic analyses of the exoproteomes revealed the presence and regulation of a GH25 muramidase and proteins exhibiting an NlpC/P60 domain that may possibly exhibit lytic activity [49]. To prove the presence of cell wall hydrolases in the supernatants of *L. hordei* TMW 1.1822 in glucose and sucrose, a zymogram analysis was performed. The stained gels containing dead cells of *M. luteus* TMW 2.96 displayed one hydrolytic band at around 110 kDa (Figure 5B) when cells were treated with glucose, indicating the presence of a cell wall active enzyme. The same result was obtained for gels containing dead *L. hordei* TMW 1.1822 cells (data not shown). However, it was not possible to assign a protein to the released exoproteomic proteins of this size.

### 3.3. Sugar Consumption and Acid Formation of Cells Grown in Either Glucose or Sucrose

**Figure 6:**
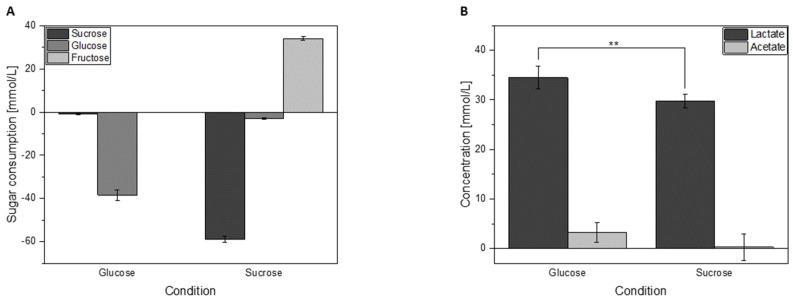
Sugar consumption (**A**) and acid formation (**B**) as quantified by HPLC analysis of culture supernatants from cells incubated in either glucose or sucrose. ** = *p* ≤ 0.01.

The proteomic experiment gave insights into the basic response of *L. hordei* TMW 1.1822 to sucrose. However, it could not show whether these proteomic changes were phenotypically visible in the sugar metabolism. Therefore, the supernatants (= exoproteome samples) were subjected to sugar and acid quantification by HPLC analysis. Within 2 h of incubation, glucose-treated cells consumed around 27% glucose (Figure 6). In sucrose-treated cells, around 77% of sucrose was consumed within 2 h of incubation. However, as the dextransucrase can extracellularly split sucrose into glucose/dextran and fructose [50], 3.0 ± 0.18 mmol/L glucose and 34.1 ± 0.78 mmol/L fructose were detectable in supernatants of sucrose-supplemented cultures (Figure 6A).

As a result of metabolic activity, glucose-treated cells produced 34.5 ± 2.3 mmol/L lactate and 3.3 ± 2.0 mmol/L acetate, while sucrose-treated cells produced 29.8 ± 1.3 mmol/L lactate and 0.3 ± 2.6 mmol/L acetate (Figure 6B). Therefore, sucrose-treated cells produced significantly (*p* < 0.01) less lactate than glucose-treated cells. Neither mannitol nor ethanol could be detected in culture supernatants.

### 3.4. Growth Characteristics of L. hordei in Different Sugars

**Figure 7:**
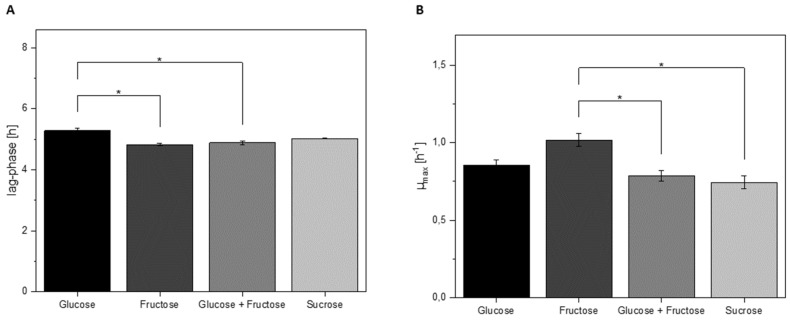
Lag phase (**A**) and maximum growth rates (µ_max_) (**B**) of *L. hordei* TMW 1.1822 in glucose, fructose, sucrose and a combination of glucose and fructose. * = *p* < 0.05.

In order to investigate the role of sucrose in the growth of *L. hordei* TMW 1.1822, general growth parameters and acidification of the cultivation media were obtained not only in glucose or sucrose but also in media supplemented with fructose or a combination of glucose and fructose. As sucrose is extracellularly degraded to fructose and glucose/dextran, all four carbohydrates were taken into account. While the lag phase of cells grown in glucose was significantly (*p* < 0.05) longer than in fructose or a mixture of glucose and fructose, sucrose did not significantly shorten the lag phase of *L. hordei* TMW 1.1822 (Figure 7A).

Similarly, maximum growth rates (µ_max_) of cultures grown in either glucose or sucrose were not significantly different. By contrast, maximum growth rates were significantly (*p* < 0.05) higher in cultures with fructose or a mixture of glucose and fructose compared to growth in sucrose (Figure 7B). The final pH levels of all four conditions were comparable (glucose 3.81 ± 0.01; fructose 3.72 ± 0.03; glucose + fructose 3.83 ± 0.02; sucrose 3.83 ± 0.00).

Furthermore, viable cells of the proteomic samples were counted in order to ensure un-biased analysis. Average viable cell counts of both groups (glucose: 8.3 ± 0.5 × 10^9^ CFU/mL; sucrose: 8.5 ± 0.8 × 10^9^) were demonstrated to be statistically not different (*p* < 0.05).

### 3.5. Screening for Dextransucrases in Other L. hordei Isolates

It was previously shown that also another water kefir isolate of *L. hordei*, namely strain TMW 1.1907, encodes for the same type of dextransucrase as strain TMW 1.1822 [16]. To investigate whether the dextransucrase is a common feature of *L. hordei* isolates, several different strains of our in-house culture collection, all isolated from water kefir, were screened for the dextransucrase gene by PCR. Additionally, the type strain *L. hordei* DSM 19519 was tested for a dextransucrase gene using degenerated primers (DSc_2900 primerset) to cover a broader spectrum of different dextransucrases. However, no PCR product was formed from the type strain’s DNA, confirming the absence of a dextransucrase gene, which was previously predicted via genome sequence analysis [36]. By contrast, all water kefir isolates tested positive for a dextransucrase gene in PCR analysis (Figure S1).

## 4. Discussion

### 4.1. Expression and Release of the L. hordei TMW 1.1822 Dextransucrase

While in *Weissella* and *Leuconostoc* species, the expression of dextransucrases was reported to be most often specifically stimulated by its substrate sucrose, many other LABs express their dextransucrases independently of this sugar [33,51,52,53,54,55]. In the present study, this could also be observed for *L. hordei* TMW 1.1822 dextransucrase, suggesting sucrose-independent expression. Furthermore, it was demonstrated by mass spectrometry that the dextransucrase is among the most abundant proteins in cells grown on glucose as well as sucrose-supplemented media. Dextransucrase is almost as abundant as common housekeeping proteins, like RNA polymerase sigma factor RpoD (Figure 2). Additionally, investigations of the exoproteomes of *L. hordei* TMW 1.1822 could demonstrate that the release of this enzyme is induced by sucrose. This was already indicated by our previous study, which suggested that intracellular dextransucrase accumulation occurs independently of the present carbon source and experiences boosted release solely in the presence of sucrose [24]. The significantly higher relative abundance of the dextransucrase in sucrose-supplemented culture supernatants compared to the related cell lysates furthermore supported the assumption of directed release in the presence of sucrose despite the absence of a known type of signal peptide (Figure 4) [24].

Several studies could also demonstrate that it is possible to obtain the *L. hordei* TMW 1.1822 dextransucrase in a buffered cell suspension, as the enzyme is freely released into the extracellular milieu due to the absence of an LPxTG membrane anchor [21,24]. The analysis of the exoproteomic MS intensities revealed that the dextransucrase was among the 20 most abundant proteins in sucrose-supplemented cultures. Therefore, it is likely responsible for most of the extracellular sucrose turnover of *L. hordei* TMW 1.1822, leading to dextran formation upon simultaneous fructose release, which is subsequently metabolized. The resulting biofilm formation may protect the microorganism against desiccation along with surface adhesion, helping *L. hordei* to gradually colonize habitats rich in sucrose, e.g., plants or fermented foods such as water kefir, once the sugar is detected. Therefore, efficient extracellular sucrose degradation upon simultaneous exopolysaccharide formation appears to be a decisive trait to subsist in such environments, as was also shown for other members of the genus *Liquorilactobacillus*, such as *L. sucicola* and *L. mali* (formerly *Lactobacillus sucicola* and *Lactobacillus mali*) [34,56,57]. This is further supported by the results from PCR analysis of several different water kefir-borne *L. hordei* strains compared to the type strain counterpart. It could be shown that all water kefir isolates encoded for a dextransucrase gene. By contrast, the type strain counterpart, isolated from malted barley, which offers a different spectrum of carbohydrates, was negative for such a gene. The *L. hordei* dextransucrase was previously shown to produce a dextran with colloidal cloud-forming properties [16,21], which has also good gel-forming properties at higher concentrations [58]. This natural trait of water kefir-borne *L. hordei* strains may thus be exploited in plant-based food and beverage fermentations, yielding clean label products with advanced sensorial and techno-functional properties.

### 4.2. Carbohydrate Metabolism in Glucose and Sucrose-Treated Cells of L. hordei

The switch from glucose to sucrose as sole carbon source led to the up-regulation of two different fructose-specific PTS systems. While one of these PTS systems belonged to the Man family (PTS*^man1^*), directing the uptake of fructose and simultaneous 6-phosphorylation, the other belonged to the Fru family (PTS*^fru^*), guiding the uptake of fructose and mannitol upon simultaneous 1-phosphorylation. This indicates enhanced uptake of fructose, which was previously released during the extracellular dextransucrase reaction (see Section 4.1, Figure 6). Correspondingly, 1-phosphofructokinase was up-regulated in sucrose, enabling the efficient processing of 1-phosphorylated fructose to fructose-1,6-bisphosphate, which is an early intermediate of glycolysis. However, another Man family PTS system (PTS*^man2^*) was significantly less present in sucrose. As the Man family PTS systems are not only specific for the uptake of fructose but also for mannose, glucose or N-acetylglucosamine, it is most likely that the PTS*^man2^* is involved in the uptake of substances that are related to glucose metabolism [59]. Furthermore, the down-regulation of 6-phosphofructokinase, which may directly feed 6-phosphorylated fructose into the glycolytic pathway, indicates that the fructose-1,6-bisphosphate pool is not primarily filled from this branch of the initial carbohydrate metabolism in sucrose-treated cells. The simultaneous down-regulation of glucose-6-phosphate-isomerase further supports this hypothesis.

**Figure 8:**
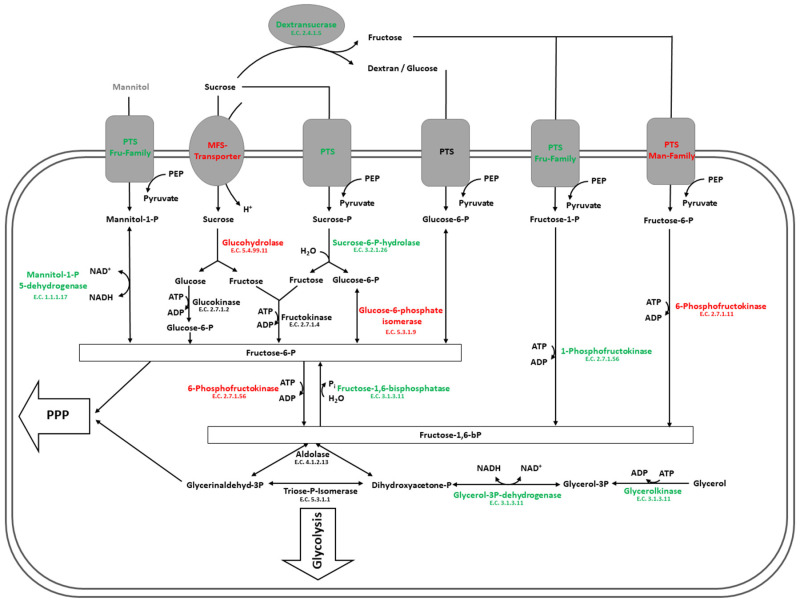
Metabolic pathways as suggested from genomic features and proteomic analysis of cell lysates of cultures incubated in either glucose or sucrose. Green color indicates up-regulation in sucrose, while red color indicates down-regulation in sucrose. PPP = pentose phosphate pathway. Part of this figure is based on Di Xu et al. [36].

Furthermore, the enzyme fructose-1,6-bisphosphatase was significantly up-regulated in sucrose, which is a central enzyme of gluconeogenesis. Therefore, it seems like *L. hordei* TMW 1.1822 fills its fructose-6-phosphate pools, which may inter alia be used in the pentose-phosphate pathway to generate reductive power and pentoses for nucleotide synthesis. This is further supported by the result that glycerolkinase and glycerol-3-phosphate dehydrogenase were additionally up-regulated in sucrose-treated cells, yielding dihydroxyacetone phosphate, which can either react during glycolysis or to glyceraldehyde-3-phosphate. Together with fructose-6-phosphate, the latter may then be used by transketolase to yield xylulose-5-phosphate and erythrose-4-phosphate, which link carbohydrate metabolism to the synthesis of other compounds, respectively, e.g., nucleotides, vitamins and certain amino acids. The possible metabolic pathways during initial carbohydrate degradation are summarized in Figure 8.

Altogether, the results relating to the effects of sucrose on the initial carbohydrate metabolism suggest that fructose is rather metabolized than glucose in sucrose-treated cells, although glucose may also be released by hydrolysis from the extracellular dextransucrase reaction.

This is further supported by the results from growth experiments in different sugars, where the lag phase of *L. hordei* TMW 1.1822 was the shortest in fructose, although the lag phase of other sugar combinations that included fructose was not significantly different. By contrast, the lag phase of cells grown in glucose was significantly longer. Furthermore, maximum growth rates were highest in fructose, while all sugar combinations that included glucose had (significantly) lower maximum growth rates, which was possibly due to catabolite repression of other sugar-converting enzymes in the presence of glucose. Additionally, the final pH after 30 h of incubation was the lowest in fructose, indicating stronger acidification and therefore enhanced metabolic activity in this sugar. Taken together, these results hint at the fructophilic nature of *L. hordei* TMW 1.1822, once more emphasizing the adaptation of this microorganism to the fruit-based fermentation of water kefir.

Although these experiments clearly showed that other sugars than glucose had an enhancing effect on growth and metabolism of *L. hordei* TMW 1.1822, this difference was not observable from cell counts in glucose and sucrose-treated cells after 2 h of incubation. However, the microorganism was grown in glucose until mid-exponential growth phase and *L. hordei* may have possibly needed some time to adapt its metabolism from glucose to sucrose. This assumption also applies for the consumption of sugars, as well as the production of acids, in the same samples. Within 2 h of incubation, 27% of glucose was consumed, while already 77% of sucrose was split (Figure 6). As *L. hordei* intracellularly accumulates its dextransucrase and releases it immediately once its substrate is detected (see Section 4.1), the microorganism is optimally prepared for rapid extracellular sucrose degradation. However, the products formed during this reaction were not consumed within 2 h of incubation. Under the assumption that sucrose is exclusively converted by the extracellular dextransucrase reaction, solely 32% of the released fructose would have been metabolized at this point in time. Moreover, significantly more lactate was formed in glucose-treated cells within 2 h of incubation, which may hint at a different metabolic route of internalized substrate in sucrose-treated cells.

Additionally, a sucrose-specific PTS system (PTS*^scr^*) was up-regulated in sucrose-treated cells, which shows that not all of the supplied sucrose is used by the extracellular dextransucrase. Within the same gene cluster, the up-regulated sucrose-6-phosphate hydrolase ensures metabolism of internalized and phosphorylated sucrose, cleaving it into glucose-6-phosphate and fructose. By contrast, a sucrose-specific MFS transporter was significantly down-regulated in sucrose, suggesting that *L. hordei* TMW 1.1822 adjusts its sucrose uptake, which may also be dependent on the concentration of this substrate (Figure 8). This may help the microorganism to avoid intracellular dextran formation by not yet released dextransucrase, which may lead to cell lysis. Within the same gene cluster, a glucohydrolase with putative α-glucosidase activity was significantly down-regulated, which may cleave un-phosphorylated intracellular sucrose into glucose and fructose. However, sucrose phosphorolysis is energetically favored over sucrose hydrolysis, as the latter reaction happens at the expense of ATP. α-glucosidases represent a group of enzymes with a large range of substrate specificities. Oligo-1,6-glucosidases also belong to this group, which are capable of dextran degradation [60]. As both differentially expressed glucohydrolases with putative α-glucosidase activity were not identifiable in exoproteomes of *L. hordei* TMW 1.1822 (see Table S2), extracellular dextran hydrolysis is unlikely. However, both enzymes may be involved in the intracellular metabolism of short-chain isomaltooligosaccharides, which are produced during the early steps of dextran formation [50]. Nonetheless, both proteins were significantly down-regulated in sucrose, which is contrary to similar enzymes in other lactic acid bacteria [33,60]. This may hint at a divergent role of this enzyme in the carbohydrate metabolism of *L. hordei* TMW 1.1822, which remains to be elucidated.

This also applies for a putative extracellular beta-fructosidase that was not significantly differentially expressed in cell lysates but was certainly more released in the presence of sucrose. Beta-fructosidases are capable of hydrolyzing sucrose and also other substrates like fructans [61]. Regarding the application of *L. hordei* TMW 1.1822 in plant-based food fermentations aiming at the in-situ production of dextran, this enzyme may appear as a competing reaction to dextran production. However, the dextransucrase was shown to be of distinctly higher abundance than the beta-fructosidase, implying that this enzyme plays solely a minor role in extracellular sucrose degradation. Furthermore, this particular beta-fructosidase exhibits a C-terminal LPxTG motif, indicating its covalent attachment to the cell surface of *L. hordei* TMW 1.1822. However, harsh cell separation techniques, such as centrifugation, may have led the enzyme to be lost from the cell wall. Moreover, this enzyme might also act as a fructan hydrolase, which would exhibit additional advantages for *L. hordei* to survive in the water kefir environment, as some of the other inhabiting microorganisms were shown to produce levan from sucrose [17]. Smaller fructooligosaccharides may then be efficiently imported by the sucrose-specific PTS system (PTS*^scr^*), as was reported for *Lactiplantibacillus plantarum* [62] (formerly *Lactobacillus plantarum* [34]).

Additionally, a gene cluster involved in the uptake and metabolism of mannitol was significantly up-regulated in sucrose (Table 1). As there was certainly no extracellular mannitol detectable in un-fermented MRS media, the PTS system (PTS*^fru^*) was in this experiment more likely utilized for fructose uptake. By contrast, the mannitol-1-phosphate 5-dehydrogenase may not solely contribute to mannitol degradation, but it may also use fructose-6-phosphate to form mannitol-1-phosphate upon NAD^+^ regeneration (Figure 8). However, no mannitol was detectable in culture supernatants of *L. hordei* TMW 1.1822, which would have been of additional nutritional value for products fermented with this microorganism, as mannitol can be applied as a low-calorie sweetener with health-promoting effects [63]. Nonetheless, the sugar alcohol is a well-known compatible solute, protecting the organism against a number of stress situations, such as high osmotic pressure. The intracellular accumulation of mannitol was, therefore, reported to maintain cell turgor at low water activity [64,65]. However, due to the lack of mannitol-1-phosphatase, *L. hordei* TMW 1.1822 is not capable of producing un-phosphorylated mannitol. Whether the intracellular accumulation of mannitol-1-phosphate has the same protecting effect in environmental stress situations remains to be elucidated. This is in good agreement with other homofermentative lactic acid bacteria, among which extracellularly detectable mannitol formation is rather uncommon [63]. Nonetheless, intracellular mannitol-1-phosphate was shown to be produced in *Lactococcus lactis* but was remetabolized upon carbohydrate depletion [66]. Furthermore, the mannitol operon was reported to be sensitive to catabolite repression. When rapidly degradable carbohydrates such as glucose are internalized by their PTS system, the mannitol operon is no longer stimulated [67,68]. Therefore, the results of the differential proteomic analysis of *L. hordei* TMW 1.1822 may rather reflect glucose-induced down-regulation than sucrose-induced up-regulation of the expression of this gene cluster.

This may also apply for the differentially expressed beta-glucoside PTS systems and phospho-beta-glucosidases (Table 1). From amino acid sequence analysis, it was not possible to derive the substrate specificity of these proteins. However, beta-glucosides are often hydrolysis products of plant material, and efficient uptake and metabolic mechanism of these substrates would not be surprising in plant-adapted *L. hordei* TMW 1.1822 [36].

Regarding the final steps of carbohydrate fermentation in *L. hordei* TMW 1.1822, two proteins belonging to the pyruvate dehydrogenase complex were significantly up-regulated in sucrose. As sucrose-treated cells produced significantly less lactate within 2 h of incubation than glucose-treated cells, enhanced degradation of pyruvate to acetyl-CoA by pyruvate dehydrogenase likely took place. Additionally, less acetate was produced in sucrose-treated cells. This suggests that the fermentation of sucrose does not require additional ATP generation upon acetate formation. However, acetyl-CoA is an important intermediate linking the central carbohydrate metabolism with fatty acid metabolism and the TCA cycle (incomplete in *L. hordei* TMW 1.1822 [36]), indicating that the impact of a switch from glucose to sucrose is not limited to the initial and central carbohydrate metabolism.

Moreover, the reduced lactate and acetate formation after sucrose treatment may additionally be explained by enhanced 2,3-butanediol formation, as butanediol dehydrogenase was significantly up-regulated in sucrose-treated cells. Together with α-acetolactate decarboxylase (E.C 4.1.1.5, BSQ_10250), encoded within the same gene cluster, butanediol dehydrogenase degrades pyruvate upon NAD^+^ recycling. This proteomic change was also observable in a previous study as a result of co-cultivation with water kefir-borne *Saccharomyces cerevisiae* TMW 3.221 and is thus not exclusively induced by sucrose treatment [69]. Therefore, the buttery and fruity aroma of 2,3-butanediol in water kefir may vice versa not solely come from co-cultivation of *L. hordei* with yeasts but also from the presence of sucrose.

Other proteins not directly involved in central carbohydrate metabolism were additionally found to be differentially expressed in the presence of sucrose compared to glucose (Table 1).

This included the up-regulation of adenylosuccinate synthase (E.C. 6.3.4.4), linking the carbohydrate metabolism with the de novo synthesis of nucleotides, and may therefore hint at enhanced DNA synthesis or transcription due to sucrose treatment.

By contrast, succinate-semialdehyde dehydrogenase (E.C. 1.2.1.24) and malonate decarboxylase (E.C. 4.1.1.88), exhibiting potential activity on succinate as well as histidinol phosphatase (E.C. 3.1.3.15), were down-regulated in sucrose-treated cells. These proteins may link the central carbohydrate with amino acid metabolism. However, this remains to be elucidated in more detail for *L. hordei* TMW 1.1822.

### 4.3. Exoproteomic Features of L. hordei TMW 1.1822 in the Presence of Sucrose

The proteomic analysis of culture supernatants of *L. hordei* TMW 1.1822 revealed that the majority of the significantly differentially released proteins were predicted to be located in the cytoplasm. Sucrose is known to be osmotically active on lactic acid bacteria [70] and may have thus led to cell lysis of at least a fraction of the culture. Moreover, as sucrose can enter cells of *L. hordei* by MFS transporters, leaving the sugar un-phosphorylated, intracellular dextran formation may cause cell lysis. However, as cell counts for glucose and sucrose-treated cells were similar after plating on agar, sucrose appeared to have no significant (osmo-)lytic effect on *L. hordei* TMW 1.1822. Solely a small fraction (~20%) of the quantified cellular proteome was also specifically quantified in the exoproteomes of glucose and sucrose-treated cells, which is an additional argument against significant amounts of cell lysis. This clearly indicates that the majority of cells of *L. hordei* stayed intact during incubation in sucrose, being in good agreement with other lactic acid bacteria, where sucrose osmotic stress is rapidly eradicated by equilibration of intra- and extracellular sucrose concentrations [71].

The comparative analysis of MS intensities supported this suggestion, as it revealed that, in both conditions, around two thirds of the proteins were of significantly different abundances in cell lysates and exoproteomes (Figure 4).

Among these proteins, the dextransucrase and the putative beta-fructosidase (*sacC*) were of distinctly different abundances in exoproteomes compared to cell lysates when cells were treated with sucrose (Figure 4) (see Section 4.1 and Section 4.2). Still, in glucose and sucrose-treated cells, proteins exhibiting an NlpC/P60 domain were actively released into the extracellular milieu, while being significantly less present in sucrose-treated cells than in glucose-treated cells. This domain is frequently found in bacterial peptidoglycan hydrolases [72]. The role of these proteins will be further discussed in Section 4.4.

Interestingly, after glucose as well as sucrose-treatment, flagellar proteins were detected in distinctly higher abundance in exoproteomes than in cellular proteomes (Figure 4), while being significantly less present in sucrose-treated cells than in glucose-treated cells. Although the species *L. hordei* was originally believed to be non-motile, it was recently shown that *L. hordei* and other *Liquorilactobacilli*, such as *L. nagelii* and *L. mali*, exhibit a complete motility operon [35,73]. This operon is also present in *L. hordei* TMW 1.1822 (BSQ40_10755–BSQ_11055). The expression of the majority of these genes may enable motility in *L. hordei* TMW 1.1822, which appeared to be regulated by the present carbon source. The decreased release of these proteins in the presence of sucrose may thus hint at reduced motility under biofilm formation conditions, as was reported for *Bacillus subtilits* [74]. Moreover, flagellar proteins of other lactic acid bacteria were reported to exhibit an immunomodulatory effect [75], which was observed to be a beneficial health effect of water kefir consumption [76].

From SEED-based analysis as well as GO enrichment analysis, it could be shown that the majority of proteins that were increasingly released in the presence of sucrose were related to protein metabolism and translation (Figure 3, Table 2). However, these proteins were not found to be among the proteins that were actively released with high confidence (z-score difference exoproteome vs. cell lysate ≥2.0) (Figure 4), although many of them were still of distinctly different abundance in exoproteomes than in cell lysates. Even though it was postulated above that cell lysis did not happen to a significant extent, when sucrose was present, this increased release of intracellular proteins may still have been an effect of high abundances of these proteins within the cellular proteomes and may thus point at a leakage of sucrose-treated cells. However, it was frequently reported that intracellular proteins, such as elongation factors, molecular chaperones (e.g., *GroL*, *DnaK*), ribosomal proteins, glycolytic enzymes (e.g., glucose-6-phosphate isomerase, glycerinaldehyde-3-phosphate dehydrogenase, phosphoglycerate mutase, enolase) and pyruvate degrading enzymes (e.g., lactate dehydrogenase), among others, can overtake other functions when released into the extracellular milieu, mostly acting as adhesion factors [77,78,79,80,81]. Glycosyltransferases, involved in exopolysaccharide synthesis from sucrose, were also shown to mediate cell aggregation and are thus responsible for the formation of floating biofilms in *Limosilactobacillus reuteri* (formerly *Lactobacillus reuteri*) [34,82]. After sucrose treatment, the exoproteome of *L. hordei* TMW 1.1822 indeed showed an increase in the multitude of such proteins, indicating that sucrose-induced biofilm formation in *L. hordei*, which was thought to be mainly composed of the extracellular polysaccharide dextran, is additionally mediated by released cytoplasmic proteins.

### 4.4. Expression and Release of Cell Wall Active Enzymes in the Presence of Sucrose Compared to Glucose

The differential proteomic analysis of cell lysates revealed up-regulated expression of a putative GH25 muramidase in sucrose-treated cells compared to glucose-treated cells. This was also observed for the analysis of exoproteomes, where it appeared to be increasingly released in the presence of sucrose. Proteins containing a GH25 muramidase domain were shown to cleave the β-(1→4) glycosidic bond between *N*-acetylglucosamine and *N*-acetylmuramic acid of bacterial peptidoglycans in *Lentilactobacillus buchneri* (formerly *Lactobacillus buchneri* [34]) [83]. However, no lytic activity could be observed from supernatants of *L. hordei* TMW 1.1822 when treated with sucrose, indicating that this enzyme has no or little hydrolytic activity against the cell wall of *L. hordei* and *M. luteus* (Figure 5). By contrast, supernatants of glucose-treated cells led to formation of a lytic zone of around 110 kDa during zymogram analysis, indicating the presence of a lytic enzyme with intra- and inter-species specificity. In the exoproteomes of glucose-treated cells, several proteins annotated as flagellum-associated murein hydrolases (*flgJ*) and proteins exhibiting an NlpC/P60 domain were found in significantly higher amounts than in sucrose-treated cells.

*FlgJ* is a cell wall active enzyme which is necessary for cell envelope remodeling during flagellar rod assembly [84], which is in good agreement with the increased release of a multitude of flagellar proteins in glucose-treated cells (see Section 4.3). However, to our knowledge, *flgJ* has not been studied in lactic acid bacteria so far, leaving its lytic role in *L. hordei* TMW 1.1822 speculative. By contrast, NlpC/P60 domain-containing proteins were characterized as γ-D-Glu-diaminoacid endopeptidases, involved in cell division and autolysis of lactic acid bacteria [85,86,87]. However, in *L. hordei* TMW 1.1822, the theoretical molecular weight of both NlpC/P60 domain-containing proteins was 41 and 44 kDa, respectively, not resembling the lytic band of 110 kDa. This may hint at the presence of an additional lytic enzyme in glucose-treated cells which may have not been quantified during proteomic analysis. Furthermore, SDS-PAGE was performed under non-denaturing conditions, which is why the lytic band may also have been caused by a multimeric enzyme.

The release of such lytic enzymes may help *L. hordei* to compete for nutrients during fermentation of water kefir, which is poor in nutrients other than sucrose. Nonetheless, these enzymes may exhibit a narrow specificity range against bacterial cell walls and may thus solely lyse certain groups of other water kefir microorganisms. However, *L. hordei* TMW 1.1822 appeared to be less competitive with other Gram-positive bacteria, including *L. hordei* itself, exhibiting no visible lytic activity after incubation in sucrose, as indicated by zymogram analysis.

## 5. Conclusions

In the present study, we extensively studied the changes in the behavior of water kefir-borne *L. hordei* TMW 1.1822 in response to sucrose by the analysis of cellular and extracellular proteomes as well as physiological tests. This showed that, in *L. hordei* TMW 1.1822, incubation in sucrose comes together with the release of its dextransucrase, which enables efficient extracellular sucrose conversion upon simultaneous fructose release and polysaccharide formation. As fructose could be demonstrated to enhance maximum growth rates and reduce the lag phase, *L. hordei* was hypothesized to be of fructophilic nature and thus optimally adapted to fruit-based fermentations. This was supported by enhanced uptake of fructose in the presence of sucrose, as indicated by the proteomic up-regulation of several fructose-specific PTS systems and degradative intracellular enzymes.

In competition with the extracellular dextransucrase reaction, an up-regulated PTS system enables sucrose uptake, while a putative extracellular beta-fructosidase may additionally split sucrose into glucose and fructose.

The exoproteome of *L. hordei* TMW 1.1822 was furthermore shown to undergo a multitude of significant changes due to the presence of sucrose. A significant (osmo-)lytic effect induced by the sucrose-supplemented medium appeared unlikely due to stable cell counts and comparative MS intensity analyses of exoproteomes and cellular proteomes. This led to the conclusion that adhesion during biofilm formation is not solely mediated by exopolysaccharides produced by the *L. hordei* dextransucrase but also by a proteinaceous component of the biofilm. Additionally, in conditions other than in the presence of sucrose, *L. hordei* TMW 1.1822 may exhibit motility due to expression and release of flagellar proteins.

Moreover, the release of an inter- and intra-species-specific lytic protein was observed in the supernatants of glucose-treated cells, which was abolished after sucrose treatment, indicating that *L. hordei* is less competitive for nutrients with its relatives and other microbes as well when incubated in sucrose.

Due to its dextran-producing abilities, *L. hordei* strains isolated from water kefir recently gained interest in relation to the fermentation of plant-based food materials. Therefore, the current study gives important insights into metabolic pathways that may occur in addition to or in competition with dextran formation. These insights lay the basis for deeper investigations, not only on the exploitability of *L. hordei* strains in food fermentations but also for further ecological studies regarding the water kefir microbiota.

## Figures and Tables

**Table 1:** Differentially expressed proteins within cellular proteomes in the presence of sucrose compared to glucose. Positive log_2_ FC values indicate up-regulated proteins in the presence of sucrose, while negative log_2_ FC values indicate down-regulation in sucrose.

#	Log_2_ FC	−Log_10_ (*p*-Value)	Function	SEED Category	FIG Identifier	Gene Loci
1	0.32	4.75	Adenylosuccinate synthase	Nucleosides and Nucleotides	fig|468911.3.peg.15	BSQ49_00075
2	0.30	4.46	Pyruvate dehydrogenase E1 subunit alpha	Carbohydrates	fig|468911.3.peg.54	BSQ49_00265
3	0.36	4.57	Pyruvate dehydrogenase E1 subunit beta	Carbohydrates	fig|468911.3.peg.55	BSQ49_00270
4	1.64	2.33	Transcriptional regulator DeoR	Carbohydrates	fig|468911.3.peg.65	BSQ49_00320
5	1.22	8.37	1-phosphofructokinase	Carbohydrates	fig|468911.3.peg.66	BSQ49_00325
6	1.17	9.08	PTS system, fructose-specific IIC component	Carbohydrates	fig|468911.3.peg.67	BSQ49_00330
7	−0.35	3.79	Crp/Fnr family transcriptional regulator		fig|468911.3.peg.84	BSQ49_00410
8	−0.41	4.27	Succinate-semialdehyde dehydrogenase		fig|468911.3.peg.97	BSQ49_00475
9	0.95	7.13	Glycerolkinase	Fatty Acids, Lipids and Isoprenoids	fig|468911.3.peg.113	BSQ49_00555
10	0.75	5.78	Glycerol-3-phosphate dehydrogenase	Fatty Acids, Lipids and Isoprenoids	fig|468911.3.peg.114	BSQ49_00560
11	1.16	5.96	PTS system, unknown specificity IIB component	Carbohydrates	fig|468911.3.peg.124	BSQ49_00610
12	1.21	7.61	6-phospho-beta-glucosidase	Carbohydrates	fig|468911.3.peg.125	BSQ49_00615
13	0.52	6.01	Sucrose-6-phosphate hydrolase		fig|468911.3.peg.158	BSQ49_00770
14	1.04	8.10	PTS system, sucrose-specific EIIBCA components	Carbohydrates	fig|468911.3.peg.159	BSQ49_00775
15	0.35	3.81	6-phospho-beta-glucosidase	Carbohydrates	fig|468911.3.peg.251; fig|468911.3.peg.1214	BSQ49_01215; BSQ49_06130
16	0.38	3.94	PTS system, unknown specificity IIA component	Carbohydrates	fig|468911.3.peg.252	BSQ49_01220
17	−0.43	2.87	Glucohydrolase (putative alpha-glucosidase activity)		fig|468911.3.peg.395	BSQ49_01980
18	−0.87	2.42	Hypothetical protein	DNA Metabolism	fig|468911.3.peg.566	BSQ49_02890
19	−0.54	2.54	Malonate decarboxylase subunit beta (biotin-independent)		fig|468911.3.peg.814	BSQ49_04135
20	−0.38	3.70	6-phospho-beta-glucosidase	Carbohydrates	fig|468911.3.peg.820	BSQ49_04165
21	−0.37	4.28	Beta-phospho-glucomutase		fig|468911.3.peg.916	BSQ49_04665
22	−0.46	6.96	6-phosphofructokinase	Carbohydrates	fig|468911.3.peg.1279	BSQ49_06450
23	−0.59	3.01	TIGR00268 family protein		fig|468911.3.peg.1648	BSQ49_08370
24	−0.45	3.23	Histidinol-phosphatase	Amino Acids and Derivatives	fig|468911.3.peg.1665	BSQ49_08455
25	−0.39	3.74	Alpha/beta hydrolase		fig|468911.3.peg.1666	BSQ49_08460
26	−0.75	8.03	Glucose-6-phosphate isomerase	Carbohydrates	fig|468911.3.peg.1667	BSQ49_08465
27	−0.32	4.61	Phosphoenolpyruvate-protein-phosphotransferase	Carbohydrates	fig|468911.3.peg.1706	BSQ49_08745
28	−0.39	5.24	6-phospho-beta-glucosidase	Carbohydrates	fig|468911.3.peg.1810; fig|468911.3.peg.1910	BSQ49_09735; BSQ49_09265
29	−0.41	4.17	PTS system, beta-glucoside specific IIABC components	Carbohydrates	fig|468911.3.peg.1911	BSQ49_09740
30	−0.49	2.41	Transcriptional antiterminator BglB	Carbohydrates	fig|468911.3.peg.1912	BSQ49_09745
31	−0.40	3.87	Transcriptional regulator (LacI family)		fig|468911.3.peg.1949	BSQ49_09920
32	−1.12	3.98	MFS transporter, sucrose-specific		fig|468911.3.peg.1950	BSQ49_09925
33	−0.46	6.18	Glucohydrolase (putative alpha-glucosidase activity)	Carbohydrates	fig|468911.3.peg.1951	BSQ49_09930
34	1.90	3.15	Fructose-1,6-bisphosphatase	Carbohydrates	fig|468911.3.peg.1958	BSQ49_09960
35	1.11	4.08	hypothetical protein		fig|468911.3.peg.1959	BSQ49_09965
36	−0.39	3.37	Dihydroneopterin aldolase		fig|468911.3.peg.1969	BSQ49_10015
37	3.56	9.55	PTS system, fructose-specific IIA component		fig|468911.3.peg.1998	BSQ49_10160
38	3.24	12.55	PTS system, fructose-specific IIB component		fig|468911.3.peg.1999	BSQ49_10165
39	3.85	4.93	PTS system, fructose-specific IID component		fig|468911.3.peg.2000	BSQ49_10170
40	3.43	9.13	PTS system, fructose-specific IIC/D component		fig|468911.3.peg.2001	BSQ49_10175
41	0.35	3.41	Butanediol dehydrogenase	Amino Acids and Derivatives	fig|468911.3.peg.2017	BSQ49_10255
42	1.42	9.64	ABC-transporter substrate-binding protein, glycerol-3-phosphate specific	Carbohydrates	fig|468911.3.peg.2080	BSQ49_10570
43	1.12	7.83	ABC-transporter ATP-binding protein	Carbohydrates	fig|468911.3.peg.2084	BSQ49_10590
44	−0.74	5.81	PTS system, fructose-specific IID component		fig|468911.3.peg.2194	BSQ49_11155
45	−0.70	3.13	PTS system, fructose-specific IIC component		fig|468911.3.peg.2195	BSQ49_11160
46	−0.76	6.01	PTS system, fructose-specific EIIAB components	Cell Wall and Capsule	fig|468911.3.peg.2196	BSQ49_11165
47	−0.79	4.83	PTS system, fructose-specific EIIB component	Cell Wall and Capsule	fig|468911.3.peg.2197	BSQ49_11170
48	−0.32	4.34	Transcription antiterminator BglG		fig|468911.3.peg.2198	BSQ49_11175
49	1.60	10.60	Mannitol-1-phosphate 5-dehydrogenase	Carbohydrates	fig|468911.3.peg.2224	BSQ49_11290
50	1.64	7.24	PTS system, fructose/mannitol specific IIA component		fig|468911.3.peg.2225	BSQ49_11295
51	1.37	4.16	transcriptional regulator	Carbohydrates	fig|468911.3.peg.2226	BSQ49_11300
52	2.73	12.28	PTS system, fructose/mannitol specific IICBA components	Carbohydrates	fig|468911.3.peg.2227	BSQ49_11305
53	0.51	5.61	GH25 muramidase (putative)			BSQ49_11795

**Table 2:** Significantly (Fisher´s exact *p*-value < 0.05) enriched gene ontologies (GO) (GO = biological processes) among differentially released proteins within exoproteomes of glucose and sucrose-treated cells. ↑ = increased release in sucrose, ↓ = decreased release in sucrose.

Regulation	GO ID	GO Term	Terms Annotated	Significant Terms	*p*-Value
↑	GO:0006412	translation	67	52	0.0375
↓	GO:0001539	cilium or flagellum-dependent cell motility	7	6	8.90 × 10^−7^
↓	GO:0030436	asexual sporulation	5	5	2.20 × 10^−6^
↓	GO:0007059	chromosome segregation	2	2	0.0067
↓	GO:0030261	chromosome condensation	2	2	0.0067

## Supplementary Materials

The following are available online at https://www.mdpi.com/2304-8158/9/9/1150/s1. Figure S1A: Results of PCR analysis using specific primers for the detection of the dextransucrase gene in water kefir-borne *L. hordei* strains TMW 1.1817 (lines 2 + 3), TMW 1.1821 (lines 4 + 5), TMW 1.1822 (lines 6 + 7), TMW 1.1907 (lines 8 + 9), TMW 1.2375 (lines 10 + 11), TMW 1.2376 (lines 12 + 13) and TMW 1.2377 (lines 14 + 15), Figure S1B: Degenerated primers were used for the detection of a dextransucrase gene in the type strain *L. hordei* DSM 19519 (lines 2 + 3), Table S1: Primer sets used for the detection of dextransucrase genes in *L. hordei* strains, Table S2: Summary of log_2_-transformed LFQ and log_10-_transformed iBAQ intensities of proteins analyzed in cellular and extracellular proteomes.
