# Supplementary material for: Living the Sweet Life: How Liquorilactobacillus hordei TMW 1.1822 Changes Its Behavior in the Presence of Sucrose in Comparison to Glucose
Source: Foods. 2020 Aug 21;9(9):1150. doi: 10.3390/foods9091150 (PMC7555045; doi:10.3390/foods9091150)
Supplement: Supplementary file 1 [file foods-09-01150-s001.zip › Suppl_table1_PCR-primer_26052020.docx]

| **Primerset** | | **Sequence 5´→ 3´** | **Annealing temperature** | **Product length** |
| --- | --- | --- | --- | --- |
| Hordei_Dsr | Forward | TTCAAGCAGCWACTAACGGM | 64.0 °C | 691 bp |
|  | Reverse | GCWCCWGCTGGCACCCAGAC |  |  |
| DSc_2906 | Forward | CAMAWGTTATYTWTCAAGGC | 52.5 °C | 315 bp |
|  | Reverse | ACCCAATCALCAATTGCYT |  |  |

Table 1: Primersets used for the detection of dextransucrase genes in *L. hordei* strains.
