# Supplementary figures and images for: Living the Sweet Life: How Liquorilactobacillus hordei TMW 1.1822 Changes Its Behavior in the Presence of Sucrose in Comparison to Glucose
Source: Foods. 2020 Aug 21;9(9):1150. doi: 10.3390/foods9091150 (PMC7555045; doi:10.3390/foods9091150)

## Slide 1
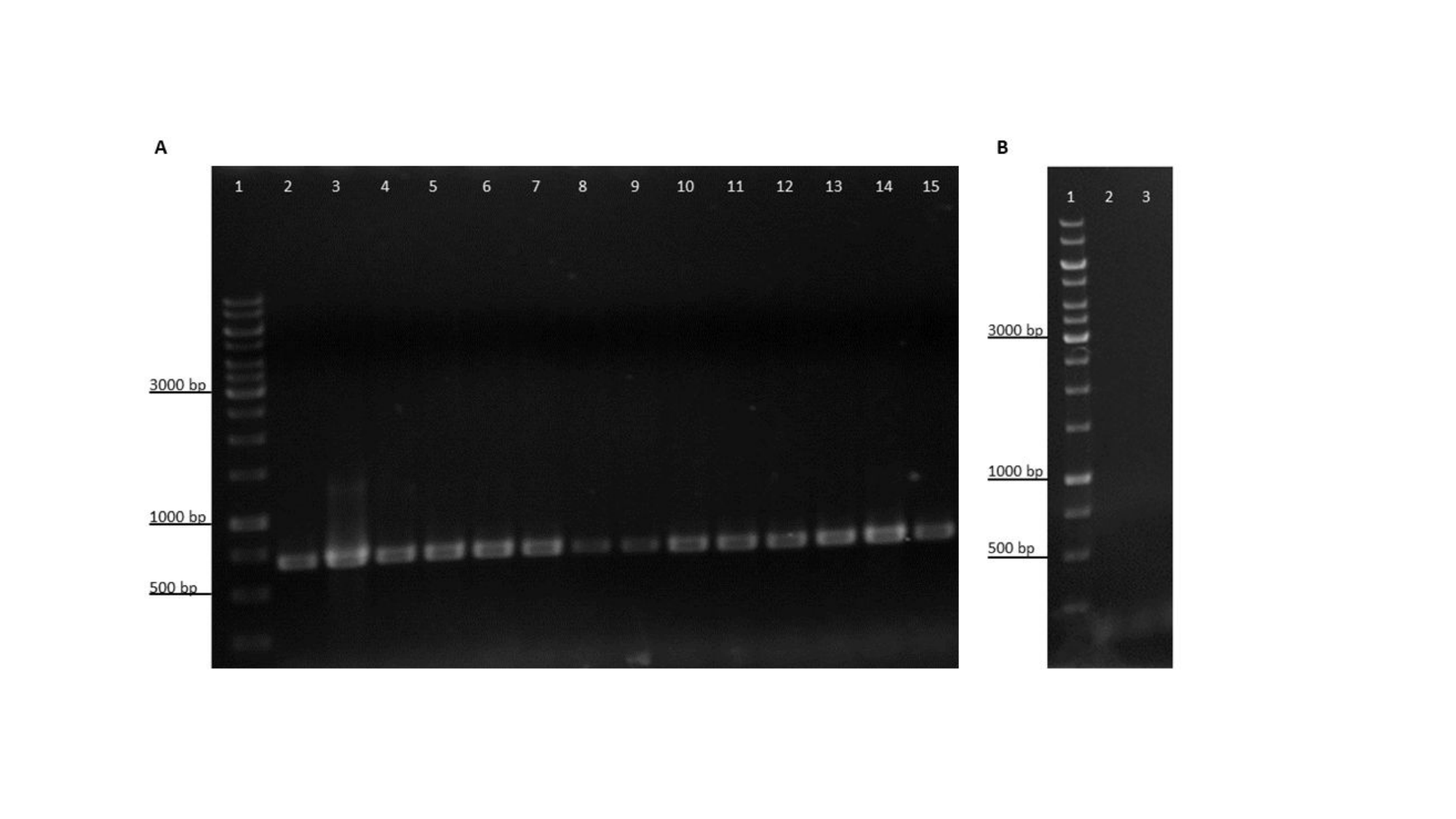

Supplement: Supplementary file 1 [file foods-09-01150-s001.zip › Suppl_fig1_PCR_25052020.pptx]
